# Establishment of a Spermatogonial Stem Cell Line with Potential of Meiosis in a Hermaphroditic Fish, *Epinephelus coioides*

**DOI:** 10.3390/cells11182868

**Published:** 2022-09-14

**Authors:** Chaoyue Zhong, Yuhao Tao, Meifeng Liu, Xi Wu, Yang Yang, Tong Wang, Zining Meng, Hongyan Xu, Xiaochun Liu

**Affiliations:** 1 State Key Laboratory of Biocontrol, Guangdong Province Key Laboratory for Improved Variety Reproduction of Aquatic Economic Animals, Institute of Aquatic Economic Animals, School of Life Sciences, Sun Yat-Sen University, Guangzhou 510275, China; 2 Southern Marine Science and Engineering Guangdong Laboratory (Zhuhai), Zhuhai 519000, China; 3 Key Laboratory of Freshwater Fish Reproduction and Development, Ministry of Education, Key Laboratory of Aquatic Sciences of Chongqing, College of Fisheries, Southwest University, Chongqing 402460, China

**Keywords:** spermatogonial stem cells, self-renewal, meiosis, hermaphrodite, orange-spotted grouper

## Abstract

Spermatogonial stem cells (SSCs) are unique adult stem cells capable of self-renewal and differentiation into sperm. Grouper is a protogynous hermaphroditic fish farmed widely in the tropical and subtropical seas. In this study, we established an SSC line derived from adult testis of orange-spotted grouper, *Epinephelus coioides*. In the presence of basic fibroblast growth factor (bFGF) and leukemia inhibitory factor (LIF), the cells could be maintained with proliferation and self-renewal over 20 months and 120 passages under in vitro culture conditions. The cells exhibited strong alkaline phosphatase activity and the characteristics of SSCs with the expression of germ cell markers, including Vasa, Dazl, and Plzf, as well as the stem cell markers Nanog, Oct4, and Ssea1. Furthermore, the cultured cells could be induced by 11-ketotestosterone treatment to highly express the meiotic markers Rec8, Sycp3, and Dmc1, and produce some spherical cells, and even sperm-like cells with a tail. The findings of this study suggested that the cultured grouper SSC line would serve as an excellent tool to study the molecular mechanisms behind SSCs self-renewal and differentiation, meiosis during spermatogenesis, and sex reversal in hermaphroditic vertebrates. Moreover, this SSC line has great application value in grouper fish aquaculture, such as germ cell transplantation, genetic manipulation, and disease research.

## 1. Introduction

Grouper, a large seawater and economic fish, is widely farmed in the tropical and subtropical seas, especially in the coastal areas of South China. Grouper is a hermaphroditic fish with a female-to-male change in its life history [1]. However, the molecular mechanism of sex reversal in hermaphrodites has not been clearly investigated. In male vertebrates, spermatogonial stem cells (SSCs) are the only adult stem cells capable of self-renewal and differentiation into sperm for transmitting genetic information to offspring [2]. However, SSCs only account for a tiny ratio of total testis cells [3,4]. It is difficult to explore the SSCs biology directly using testis tissue, or obtain a large number of SSCs for in vitro manipulations, such as germ cell transplantation and genetic manipulation. Therefore, the long-term culture system of SSCs presents remarkable advantages for studies on the molecular mechanisms behind SSCs self-renewal and differentiation, and the applications of germline stem cells [5,6]. Likewise, the establishment of a grouper SSC line will provide unique insights to understand the mechanism of sex reversal in hermaphrodites, as well as to bring a stem cell tool for grouper breeding.

The mammalian SSC line with an infinite multiplication capacity can be obtained by cellular immortalization [7,8,9]. The mouse (*Mus musculus*) SSCs after immortalization express only 10 of 36 spermatogonial markers [10]. The immortalized SSC line of goat (*Capra hircus*) could produce sperm-like cells in vitro by retinoic acid stimulation [11]. However, no report shows the generation of fertile sperm from immortalized SSC lines so far. Immortalization might have adverse effects on the fertility of SSCs and greatly limits the application value of SSC lines in animal breeding. Therefore, it is necessary to establish the nonimmortalized SSC line with normal fertility for an in-depth study of SSCs self-renewal and spermatogenesis.

The self-renewal of SSCs without immortalization depends primarily on some cytokines, such as glial cell line-derived neurotrophic factor (GDNF), basic fibroblast growth factor (bFGF), leukemia inhibitory factor (LIF), and epidermal growth factor (EGF) [12,13]. The neonatal mouse SSC line proliferates over 2 years with GDNF, bFGF, LIF, and EGF, and gives rise to the generation of offspring via transplantation into the recipient testis [14,15]. Intriguingly, an adult mouse SSC line can also be propagated for more than 14 months with LIF and EGF [16]. The hamster (*Mesocricetus auratus*) SSC line could be cultured for at least 1 year with GDNF, bFGF, and EGF [17]. The SSC lines of rat (*Rattus norvegicus*) and bovine (*Bos tarurs*) continuously proliferate in the presence of GDNF and bFGF [18,19]. Human (*Homo sapiens*) SSCs can survive in vitro for 7 months in the effect of GDNF, EGF, and LIF [20]. Tree shrew (*Tupaia belangeri*) SSC line is passaged over 50 generations with the medium containing GDNF, bFGF, EGF, and LIF [21].

Being lack of a suitable culture system to maintain the continuous propagation of fish SSCs, the establishment of fish SSC lines is limited [6]. The SSCs of swamp eel (*Monopterus albus*), a hermaphroditic fish, maintain survival for up to 30 days with the addition of bFGF and LIF [22]. Rohu (*Labeo rohita*) SSCs are cultured for 2 months in the effect of insulin and GDNF [23]. In the medium supplemented with insulin-like growth factor-1, bFGF, and GDNF, zebrafish (*Danio rerio*) SSCs only survive for at most 3 months and differentiate into fertile sperm after transplantation into the recipient testis [24]. GDNF can effectively support the in vitro propagation of dogfish (*Scyliorhinus canicula* L.) SSCs for at least 5 months [25]. At present, the fish SSC lines have been only reported in two species. Originating from adult testis of medaka (*Oryzias latipes*), the SSC line SG3 can proliferate over 2 years in the ESM medium containing bFGF [26]. Additionally, the in vitro recapitulation of spermatogenesis in SG3 can be entirely accomplished up to the formation of motile sperm. Recently, the SSC line of another small freshwater fish, the hook snout carp (*Opsariichthys bidens*), was reported to be established using the same ESM medium [27].

The cultured SSCs would provide a valuable tool to investigate spermatogenesis in vitro. Under the stimulation of stem cell factor (SCF) and/or retinoic acid, the cultured SSCs of human and mouse produce haploid male germ cells [28,29,30,31]. Moreover, the mouse SSC line is capable of generating fertile sperm through the organ culture method [32,33]. Interestingly, the mouse SSCs can be induced into haploid oocytes under the culture condition of LIF, EGF, B27, insulin, and follicle-stimulating hormone [34]. In the co-culture system of buffalo (*Bubalus bubalis*) SSCs and Sertoli cells, the formation of sperm-like cells needs the involvement of retinoic acid, testosterone, and follicle-stimulating hormone [35]. In fish, such as zebrafish and Japanese eel (*Anguilla japonica*), 11-ketotestosterone (11-KT) and testosterone are often employed to induce in vitro spermatogenesis [36,37,38]. Catfish (*Clarias batrachus*) SSCs after 2 months of culture spontaneously differentiate into motile sperm that fertilizes egg and gradually grows up to fingerling [39]. After being co-cultured with Sertoli cells, zebrafish SSCs can generate functional sperm that gives rise to fertile adult fish [40]. In a word, the cultured SSCs have great potential for applications in genetic manipulation in fish breeding through producing sperm in vitro.

At present, the SSC line has not yet been reported in hermaphroditic vertebrates and marine fish. In this study, we identified and established an SSC line derived from adult testis of orange-spotted grouper, *Epinephelus coioides*. Subsequently, the effect of bFGF, LIF, and SCF on the cultured cells was examined in terms of self-renewal, differentiation, and gene expression. Moreover, the meiotic potency of the cells was determined by examining the expression of meiotic marker genes and cell morphology after the 11-KT treatment. Our study would facilitate exploiting germ cells in fish breeding biology and the findings of this study would pave a way for further intensive investigations on the molecular mechanisms behind SSCs self-renewal, spermatogenesis, and sex reversal in hermaphroditic vertebrates.

## 2. Materials and Methods

### 2.1. Ethic Statement

All animal experiments were carried out under the guidelines and approval of the Institutional Animal Care and use Committee of Sun Yat-Sen University (Approval Number SYSU-IACUC-2021-B0494).

### 2.2. Fish

Orange-spotted groupers were collected from the Marine Fisheries Development Center of Guangdong Province, Huizhou 516081, Guangdong, China. The adult male groupers, about 65 cm in length and 5 kg in weight, were confirmed by squeezing milky semen out of their belly. The grouper fish was euthanized with 30 mg/L eugenol (Solarbio, Beijing, China) before being sacrificed.

### 2.3. Histological Examination

The testis was cut into fragments about 5 mm^3^. The testis fragments were fixed for 24 h in Bouins’ solution (Solarbio), dehydrated with serial grades of ethanol, and treated with xylene. After being embedded in paraffin, the testis was serially sectioned at 5 µm and then stained with hematoxylin and eosin. The histology was photographed by a Nikon Ti2-U microscopy (Nikon, Tokyo, Japan).

### 2.4. Cell Culture

The fresh testis was washed 3 times with PBS containing Pen/Strep (100 units/mL penicillin, 100 µg/mL streptomycin; Gibco, Waltham, MA, USA), then cut into tiny pieces about 1 mm^3^. The testis pieces were digested with 1 mg/mL collagenase IV (Gibco) for 20 min and then 0.25% trypsin (Invitrogen, Carlsbad, CA, USA) for 5 min. After being centrifuged at 500× *g* for 5 min, cells’ pellets and residual pieces were resuspended in medium and pipetted into gelatin-coated 25 cm^2^ bottles (Corning, New York, NY, USA) for cultivation at 28 °C. Medium was changed every 2 days during the first week. Cells were subcultured every 3 to 5 days with a split ratio of 1:3. The medaka SG3 was provided by Prof. Hongyan Xu [26]. Cells were imaged by a Leica DMI8 microscope (Leica, Wetzlar, Germany).

Since ESM medium can support the long-term proliferation of medaka SG3 [26], medaka embryonic stem cell lines [41,42,43], and an ovarian stem cell line from soft-shell turtle [44], we used this medium for culturing grouper testicular cells. Formula of ESM medium with minor modifications: Dulbecco’s modified Eagle medium containing 20 mM Hepes (Solarbio), 15% fetal bovine serum (Gibco), Pen/Strep (100 Units/mL penicillin, 100 µg/mL streptomycin; Gibco), 10 ng/mL recombinant human bFGF (Novoprotein, Suzhou, China), 10 ng/mL recombinant human LIF (Novoprotein), 1 mM nonessential amino acids (Gibco), 1 mM sodium pyruvate (Gibco), 2 mM glutamine (Gibco), 2 nM sodium selenite (Sigma, Burlington, MA, USA), 1% seabass serum, medaka embryo extracts (about 2.5 embryos per mL), and 55 µM 2-mercaptoethanol (Invitrogen), finally adjusted to PH 7.5 with 10 M sodium hydroxide solution.

### 2.5. Single-Cell Cloning by Serial Dilution

The single-cell cloning by serial dilution was performed as described with minor modifications [10,45]. Briefly, the mixed testis cells at passage 40 were trypsinized and diluted to 5 × 10^3^ to 1 × 10^4^ cells/mL. All wells in 96-well plates (Corning) were added with 100 µL ESM medium in advance, except the first well at the top-left corner which was added with 200 µL cell suspension later. An amount of 100 µL cell suspension was transferred from the first well to the second well in the vertical direction and mixed by pipetting. Repeat dilutions down the entire column. An amount of 50 µL cell suspension in each well in the first column was also transferred to the second column. Repeat dilutions down the final column. After 2 to 3 days of culture, the wells only containing a single colony would be marked out. After 7 to 12 days of culture, these marked single colonies would be expanded in bottles, and then repeated the above steps up to 4 rounds for establishing stable cell lines.

### 2.6. Alkaline Phosphatase Staining

Following the previous reports [26,44], alkaline phosphatase staining was performed in cells. Being propagated to about 70% confluence in a 12-well plate, cells were fixed in 4% paraformaldehyde for 15 min. After being washed twice with 0.2 M Tris-HCl solution (Solarbio), cells were incubated in BCIP/NPT Stock Solution (Roche, Mannheim, Germany) containing 0.188 mg/mL BCIP and 0.375 mg/mL NBT in darkness at room temperature for 6 h. Cells were washed twice with PBS and imaged with a microscope (Leica).

### 2.7. Chromosome Analysis

The cells were incubated with 2 µg/mL colcemid at 28 °C for 6 h. After trypsinization, cells were centrifuged at 500× *g* for 5 min. The cells’ pellet was resuspended in 0.0375 M KCl for 45 min, followed by 2 rounds of centrifugation and fixation with cold methanol-acetic acid (3:1 *v*/*v*) for 15 min. The fixed cell suspension was dropped onto cold glass slides, air-dried, and stained with 10% Giemsa solution (Solarbio) for 10 min. Chromosomes were counted and imaged with a microscope (Leica).

### 2.8. Western Blotting

Total protein was extracted from gonads and cultured cells with RIPA Lysis Buffer (Beyotime, Beijing, China), and then mixed with SDS-PAGE Sample Loading Buffer (Beyotime). After being boiled for 5 min, 15 µL protein buffers were loaded into lanes, electrophoresed through 10% SDS-polyacrylamide gels, and electroblotted onto polyvinylidene difluoride membranes (Merck Millipore, Billerica, MA, USA) via an electroblotter (Biorad, Hercules, CA, USA). The membranes were blocked with 5% BSA (Sigma) in TBS buffer (Solarbio) added with 0.1% Tween 20 (Beyotime) for at least 1 h. After washing with TBS, the membranes were incubated with antibodies (1:1000 dilution in TBS) for 2 h. Then, the membranes were washed with TBS and incubated with HRP-conjugated goat anti-rabbit IgG (Bioss, Beijing, China) or HRP-conjugated goat anti-mouse IgG (Bioss) (1:2000 dilution in TBS) for 2 h. After the membranes were washed, protein blots were colored with the Enhanced HRP-DAB Substrate Colorimetric Kit (Tiangen, Beijing, China) and imaged with a Tanon-1600 image system (Tanon, Shanghai, China).

The rabbit polyclonal Dazl and Nanog antibodies of medaka were provided by Prof. Hongyan Xu [44]. The rabbit polyclonal Piwi and Dmc1 antibodies of medaka were also provided by Prof. Hongyan Xu (unpublished data). The rabbit polyclonal Sycp3 antibody of Nile tilapia was provided by Prof. Jing Wei in School of Life Sciences of Southwest University (unpublished data). The rabbit polyclonal Amh antibody of orange-spotted grouper was provided by Dr. Yulong Han [46]. The anti-Ssea1 and anti-PCNA antibodies were purchased from Proteintech with product codes 19497-1-AP and 10205-2-AP. The anti-β-Actin antibody was bought from Merck Millipore with product codes MAB 1501.

### 2.9. Total RNA Extraction and PCR

Total RNA was extracted from gonads and cultured cells with TRIzol reagent (Invitrogen). RNA quality was evaluated via agarose gel electrophoresis. The cDNA was synthesized with 1 µg total RNA using ReverTra Ace qPCR RT Master Mix with gDNA Remover (Toyobo, Osaka, Japan), referring to the manufacturer’s manual. Reverse transcription-polymerase chain reaction (RT-PCR) was implemented with KOD One PCR Master Mix-Blue (Toyobo). The RCR procedure was as follows: initial denaturation at 94 ° for 2 min, followed by 35 cycles of 5 s at 98 °C, 5 s at 60 °C, and 1 or 2 s at 68 °C, and finally 68 °C for 2 min. After agarose gel electrophoresis, the desired bands were photographed with an image system (Tanon), and identified by DNA sequencing in Sangon Biotech Company. The real-time quantitative PCR (RT-qPCR) was carried out on a Roche LightCycler 480 System (Roche Diagnostics, San Francisco, CA, USA) with SYBR Green Realtime PCR Master Mix (Toyobo). The RT-qPCR procedure is as follows: 30 s at 95 °C, followed by 40 cycles of 5 s at 95 °C, 5 s at 58 °C, and 15 s at 72 °C, with a final step of 15 s at 95 °C and 30 s at 60 °C. The *β-actin* was used as a reference gene. Primers are listed in Table 1.

### 2.10. ISH

Fresh testis was cut into fragments about 5 mm^3^ and fixed with 4% paraformaldehyde at 4 °C overnight, dehydrated and rehydrated with gradient methanol, and then immersed in 30% (*w*/*v*) sucrose at 4 °C overnight. The fragments were embedded in the Tissue-Tek OCT compound (SAKURA Tissue-Tek, Atlanta, GA, USA), and then sectioned at 4 µm with a Leica CM1950 frozen microtome (Leica). Probes were synthesized according to the operation guide of DIG RNA Labeling Mix (Roche). The sample slides were hybridized with 1 µg/mL DIG probes in seal boxes at 65 °C overnight. Following hybridization, the slides were washed with washing buffer (Roche) and blocked with Blocking Reagent (Roche) for at least 1 h. Probe signals were developed with an AP-conjugated anti-DIG antibody (Roche; diluted 1:2000) and colored with NBT/BCIP Stock Solution (Roche). Photographs were imaged by a microscope (Leica). Probe primers are listed in Table 1.

### 2.11. Fluorescent Immunostaining

Frozen testis sections and cultured cells were fixed with 4% paraformaldehyde for 10 min and washed twice with PBS. Later, they were blocked with 5% goat serum (Gibco) for at least 1 h and incubated with antibodies for 2 h (1:200 dilution in PBS). After being washed with PBST containing 0.1% Tween 20 (Beyotime), they were incubated with HRP-conjugated goat anti-rabbit IgG (Bioss) or HRP-conjugated goat anti-mouse IgG (Bioss) (1:2000 dilution in PBS) for 2 h. Antibody signals were colored using the TSA Plus Fluorescence Systems (PerkinElmer Life Science, Waltham, MA, USA). Nucleus was stained by propidium iodide. Photographs were imaged by a Zeiss SML800 laser scanning confocal microscope (Zeiss, Jena, Germany) or a Leica TCS SP5 laser scanning confocal microscope (Leica).

### 2.12. Effects of Different Cytokine Combinations on Cells

ESM media were supplemented with 1 to 3 kinds of cell factors at different combinations and the same concentration of 10 ng/mL, including recombinant human bFGF (Novoprotein), recombinant human SCF (Novoprotein), and recombinant human LIF (Novoprotein). ESM medium without cell factor was used as a negative control. Cells were seeded evenly in eight 12-well plates (Corning) filled with different media and at a concentration of about 5 × 10^4^ cells per well. Cells in three wells of each 12-well plate were trypsinized and counted at disparate 1, 3, 5, and 7 days using a cell counter (Countstar, Shanghai, China). Cells cultured in different media for 7 days were photographed with a microscope (Leica) and analyzed by RT-PCR and RT-qPCR. Primers are listed in Table 1 and Table 2.

### 2.13. Induction of Cell Differentiation

The induced ESM medium contained 5 ng/mL bFGF, 5 ng/mL LIF, and 10^−6^ mol/L 11-KT. The control ESM medium contained 5 ng/mL bFGF and 5 ng/mL LIF. Cells were cultured with the induced ESM medium or the control ESM medium. The media were changed every 2 days. Cells were photographed with a microscope (Leica). The cells at disparate 1, 3, 6, and 9 days were collected for the RT-qPCR analysis. Primers are listed in Table 2 and Table 3.

### 2.14. Statistical Analysis

The data in RT-qPCR analysis and cell growth rate were displayed as the mean values ± SEM of three samples. Statistical analysis was performed by one-way ANOVA and Student’s *t*-test. A probability level less than 0.05 (*p* < 0.05) was considered statistically significant. All statistics were implemented using GraphPad Prism version 5.0 (GraphPad Software, San Diego, CA, USA).

## 3. Results

### 3.1. Cellular Localization of Related Germ Cell Markers in Adult Testis

ISH and fluorescent immunostaining were performed in the adult testis of orange-spotted grouper for confirming the cellular localization of related germ cell markers. The *ly75* mRNA existed in spermatogonia and spermatocytes but not in spermatids (Figure S1A). The *thy1* was limited to all spermatogenic cells (Figure S1B). The *dmc1* was detected in spermatocytes, spermatids, and differentiated spermatogonia, but weakly in undifferentiated spermatogonia (Figure S1C). The sense probes of *ly75*, *thy1*, and *dmc1* resulted in no signals in testis (Figure S1D–F). The Piwi protein was detected in spermatogonia, while slight or absent in spermatocytes and spermatids (Figure S2A). The Dazl was concentrated in nuclei and the cytoplasm of perinuclear area in germ cells (Figure S2B). The Ssea1 was mainly detected in spermatogonia and spermatocytes but barely in spermatids (Figure S2C). The Nanog was distributed in spermatogonia and the cytoplasm of spermatocyte and spermatid (Figure S2D). The PCNA was detected in the nuclei of spermatogonia and spermatocytes, but weakly in spermatids (Figure S2M). Besides, except being found in the cytoplasm of all spermatogenic cells, the Sycp3 and Dmc1 signals were detected as one to three spots in the nuclei of spermatogonia, a faint spot in the nuclei of some spermatocytes, and an intensive spot in the nuclei of spermatids, but undetectable in the nuclei of spermatozoa (Figure S2N,O). Sycp3 is also detected as dot signals in the nuclei of spermatogonia and diffused in the cytoplasm of some germ cells in zebrafish [40]. Nuclei were counterstained with propidium iodide (PI) (Figure S2E–H,P–R). Merge images were shown in Figure S2I–L,S–U. In a word, these selected genes and antibodies could be used as the biomarkers to label the germ cells at different stages in the adult testis of orange-spotted grouper, and to identify the cultured testis cells.

### 3.2. Establishment of an SSC Line Derived from Adult Testis of Orange-Spotted Grouper

**Figure 1:**
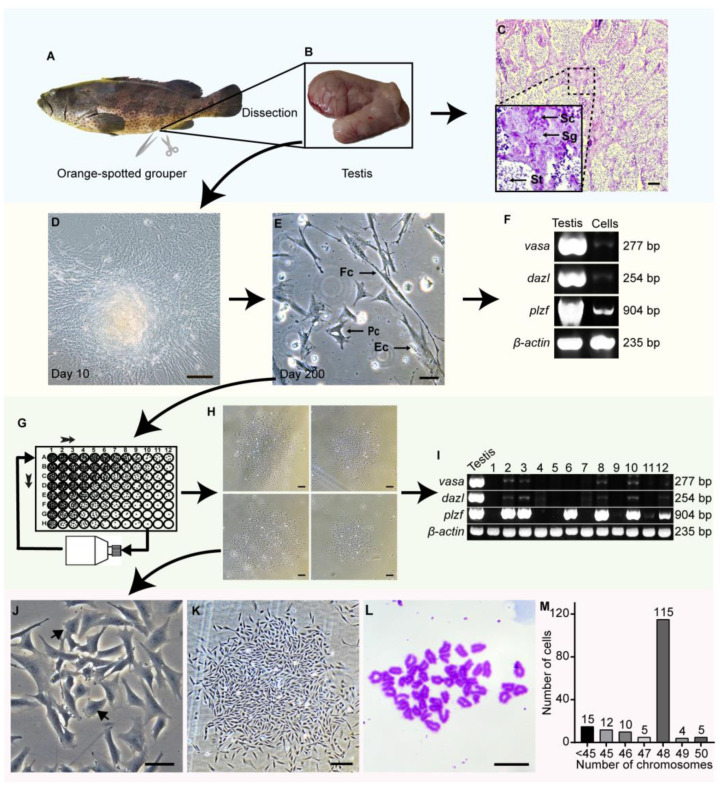
Establishment of an SSC line in orange-spotted grouper. (**A**) The fish image of orange-spotted grouper. (**B**) The testis was dissected from male orange-spotted grouper. (**C**) Histological structure of testis and the magnification of the framed area showing spermatogenic cells at different stages during spermatogenesis. (**D**) The primary cell culture on the 10th day. (**E**) Morphologies of testicular cells after 200 days of culture. (**F**) Expression of germ cell genes *vasa*, *dazl*, and *plzf* in testis and the testicular cells cultured over 200 days. The *β-actin* was used as an internal control. (**G**) Diagram of isolating single-cell colonies from the cultured testicular cells. The short arrow represents the order of serially diluting cell suspension in a 96-well plate. The dot in the wells represents a single colony. The single colony in the wells is expanded in a culture bottle for the next round of purification. (**H**) Colonies of stable testicular cell lines after four rounds of single colony selection and expansion. (**I**) Expression analysis of *vasa*, dazl, and *plzf* in testis and 12 stable testicular cell lines at passage 70. (**J**) Morphology of a putative SSC line named GPT with expressions of *vasa*, *dazl*, and *plzf*, and its nucleus with apparent nucleoli (Arrows). (**K**) A single colony of GPT cells at passage 99 and day 516. (**L**) Diploid karyotype of 48 chromosomes of GPT cells. (**M**) Chromosome number distributions in 166 metaphases of GPT cells. Sg, Spermatogonia; Sc, Spermatocyte; St, Spermatid; Fc, Fibroblast-like cell; Ec, Epithelial-like cell; Pc, polygonal-like cell. Scale Bars: 200 µm in (**C**,**H**,**K**); 100 µm in (**D**); 50 µm in (**E**,**J**); 5 µm in (**M**).

Because orange-spotted grouper during the sex reversal period has a bisexual gonad containing both male and female germ cells [1], it is necessary to check the gonadal development of the dissected grouper. The orange-spotted grouper was dissected for gonads collection (Figure 1A,B), which would be examined by histological analysis through the paraffin section. Histological analysis showed that the testis had plentiful male germ cells at different development stages including spermatogonia (Figure 1C), suggesting that the testis tissue could be used for cell culture. Testis pieces were cultured with the ESM medium containing bFGF (Figure 1D). During 200 days of culture, the testicular cells with various morphologies were observed, including fibroblast-like cells, epithelial-like cells, and polygonal-like cells (Figure 1E). Nonetheless, the testicular cells expressed *vasa* and *dazl* weakly, as well as *plzf* clearly (Figure 1F). Therefore, it was deduced that the cultured cells should contain some SSCs.

Since the SSCs in a stable culture state possess the capability to expand clonally [14,26], we tried to isolate the infrequent SSCs by single-cell cloning (Figure 1G). Single colonies of all epithelial and fibroblast-like cells and many polygonal-like cells would gradually die out after one to two rounds of unicell clonal proliferation, whereas 12 stable cell lines were derived from a small minority of polygonal-like cell colonies after four rounds of clonal expansion (Figure 1H). Interestingly, the five polygonal-like cell lines were able to express *vasa* and *dazl* weakly, as well as *plzf* strongly (Figure 1I). One of the five cell lines was designated as the germ stem cell line of protogynous testis (GPT). When ESM medium was supplemented with bFGF only, GPT cells consisted of many polygonal-like cells and some differentiated epithelial-like cells (Figure 1J). The polygonal GPT cells flattened their cytoplasm about 20 to 25 µm in diameter and had a large nucleus of about 10 µm (Figure 1J). GPT cells at passage 99 still had a strong capability to form large unicell colonies with about 20 days of proliferation (Figure 1K and Figure S3). Additionally, a major amount of GPT cells kept a normal diploid with 48 chromosomes (Figure 1L,M).

**Figure 2:**
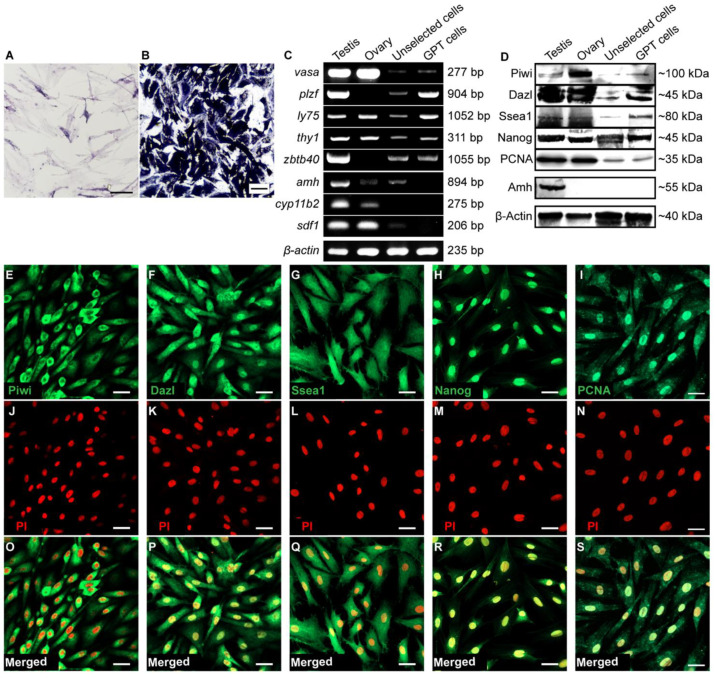
Expression of germ cell markers and pluripotency markers in GPT cells. (**A**) Alkaline phosphatase staining in the testicular cells cultured over 200 days. (**B**) Alkaline phosphatase staining in GPT cells. (**C**,**D**) Gene expression analysis in GPT cells via RT-PCR and western blotting. The cDNA and protein samples included testis, ovary, the testicular cells cultured over 200 days (unselected cells), and GPT cells. The *β-actin* was used as an internal control. (**E**–**I**) Fluorescent immunostaining analysis of Piwi, Dazl, Ssea1, Nanog, and PCNA in GPT cells. (**J**–**N**) Nucleus was counterstained with propidium iodide (PI). (**O**–**S**) Merge images. Scale Bars: 50 μm in A and B, 20 μm in (**E**–**S**).

Alkaline phosphatase exists in many kinds of stem cells, including germ stem cells [26,44,47]. In the cultured testicular cells, only a few cells were positive for alkaline phosphatase staining (Figure 2A). After four rounds of clonal expansion, the cell line GPT was strongly positive for alkaline phosphatase staining (Figure 2B). Furthermore, we identified the expression of germ cell marker genes in GPT cells. Vasa, Dazl, and Piwi are the widely accepted germ cell-specific markers and also limited to germ cells in orange-spotted grouper [48]. In orange-spotted grouper, the SSC-specific marker Plzf is only expressed in spermatogonia, and Zbtb40 is specifically limited to spermatogenic cells [49]. Ly75 is a highly conserved fish mitotic germ cell marker [50,51]. Thy1 is an SSC marker that can be used for fish SSCs enrichment [21,23]. The stem cell pluripotency markers Ssea1 and Nanog exist in germline stem cells [23,44,52]. The cell proliferation marker PCNA plays an important role in self-renewal [53] and exists in cultured germline stem cells [44,52]. Compared to the unselected testicular cells, GPT cells expressed *ly75*, *thy1*, *zbtb40*, *star*, Dazl, Ssea1, and Nanog at a higher level, whereas expression of *vasa* mRNA, PCNA, and Piwi protein were still weak (Figure 2C,D). Amh protein is strictly restricted to Sertoli cells in testis [46,54]. The *cyp11b2* mRNA is principally expressed in Leydig cells in testis [55,56]. The *sdf1* mRNA is highly expressed in Stromal cells in testis [57,58]. GPT cells did not express mRNAs of genes *amh*, *cyp11b2*, *sdf1*, and Amh protein (Figure 2C,D). In the ovary and the unselected testicular cells, *amh* mRNA could be weakly detected, but its protein was undetectable. Therefore, GPT cells should be not contaminated with the three types of testis somatic cells. The germ cell markers in GPT cells were further visualized via fluorescent immunostaining. Piwi was distributed in cytoplasm (Figure 2E). Dazl was detected in nucleus and the cytoplasm of perinuclear region (Figure 2F). Ssea1 diffused in cells (Figure 2G). The Nanog and PCNA were restricted to nucleus of cells (Figure 2H,I). Nuclei of GPT cells were counterstained with PI (Figure 2J–N). Merge images were shown in Figure 2O–S. Taken together, an SSC line, GPT was established from adult testis of orange-spotted grouper via single-cell clonal expansion and the ESM medium containing bFGF.

### 3.3. Effects of bFGF, LIF, and SCF on the Morphology and Gene Expression of GPT Cells

**Figure 3:**
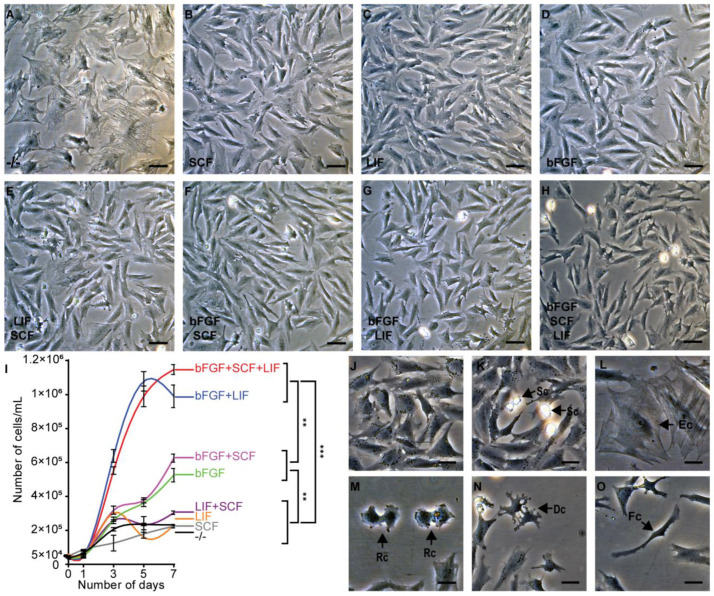
Effect of different cytokine combinations on the morphology and growth rate of GPT cells during 7 days of culture. (**A**) All GPT cells differentiated into large epithelial-like cells in medium without bFGF, LIF, and SCF (-/-). (**B**,**C**,**E**,**F**) GPT cells generated many epithelial-like cells under the condition of 4 combinations LIF, SCF, LIF and SCF, and SCF and bFGF. (**D**) GPT cells consisted of epithelial-like cells and polygonal-like cells in the presence of bFGF. (**G**) GPT cells consisted of most polygonal-like cells and a few epithelial-like cells in the presence of LIF and bFGF. (**H**) Compared with the bFGF and LIF combinations, the bFGF, LIF, and SCF group had no obvious effect on cell morphology. (**I**) Growth curve of GPT cells under different cytokine combinations. Cell number was shown as the mean ± SEM of three samples and the values with asterisks were significantly different (** *p* < 0.01, and *** *p* < 0.001). Statistical analysis was performed by one-way ANOVA and Student’s *t*-test. (**J**) Most GPT cells treated with bFGF and LIF showed a polygonal-like cell shape about 20 µm in diameter. (**K**–**O**) GPT cells generated a few cells with diverse morphology (Arrows) in the coexistence of LIF and bFGF. Sc, spherical cells (different sizes); Ec, Epithelial-like cells; Rc, Round cells (under a state of cell division); Dc, Dendritic-like cells; Fc, Fibroblast-like cells. Scale Bars: 50 μm in (**A**–**H**); 20 μm in (**J**–**O**).

To investigate the effect of different cytokines on the self-renewal of the GPT cells, we prepared a variety of media containing different combinations of cytokines. Withdrawing bFGF, LIF, and SCF, all GPT cells quickly differentiated into epithelial-like cells with a very large adherent area during 7 days of culture (Figure 3A). Under the influence of SCF or LIF, GPT cells showed an epithelial-like cell shape (Figure 3B,C). GPT cells incubated with supplemental bFGF produced epithelial-like cells and polygonal-like cells (Figure 3D). In presence of LIF and SCF, or bFGF and SCF, GPT cells generated many epithelial-like cells (Figure 3E,F). Unexpectedly, in the coexistence of bFGF and LIF, GPT cells consisted of most polygonal-like cells with a lesser adherent area of around 20 µm in diameter, as well as fewer epithelial-like cells (Figure 3G). The addition of SCF into the medium containing bFGF and LIF did not make GPT cells acquiring more obvious morphologic alteration (Figure 3H). Additionally, bFGF was able to promote cell propagation, and LIF could also significantly accelerate cell growth rate in the presence of bFGF, while other cytokine combinations had no prominent effects on cell growth (Figure 3I). In the coexistence of bFGF and LIF, GPT cells mainly exhibited polygonal (Figure 3J), and had a sufficient differentiation capacity for generating a few cells with diverse morphology (Figure 3K–O), such as spherical cells at different sizes, round-like cells, and dendrite-like cells. During 35 days of culture in the ESM media, lacking bFGF but containing SCF and/or LIF, all GPT cells finally were transformed into large epithelial-like cells and disappeared gradually (Figure S4). Compared with other combinations, the bFGF and LIF groups with or without SCF could significantly promote cell proliferation, and maintain the cell morphology and stem cell potential of the GPT line.

**Figure 4:**
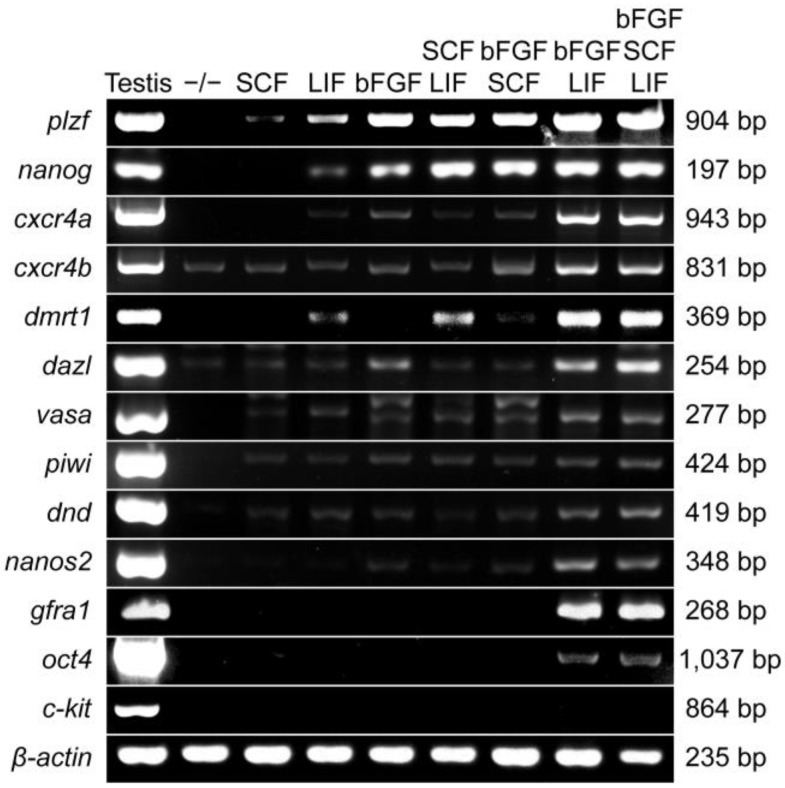
Expression analysis of germ cell marker genes in GPT cells treated with different cytokine combinations. The cDNA samples included testis and the GPT cells cultured with different cytokine combinations or without bFGF, LIF, and SCF (-/-). In the -/- combination, the GPT cells almost did not express any of the listed germ cell marker genes, except for *cxcr4b* and *dazl*. In the bFGF and LIF combinations supplemented with or without SCF, almost all listed germ cell marker genes could be detected in the GPT cells, except for *c-kit*. In the remaining combinations, the GPT cells expressed only some of the listed germ cell marker genes. The *β-actin* was used as an internal control.

Furthermore, we examined that the cytokines affected the expression of germ cell marker genes in the GPT cells (Figure 4). Cxcr4 is necessary for the maintenance of germline stem cells [58,59]. Nanos2 is a germline stem cell-specific marker in orange-spotted grouper [60]. Dnd is a vertebrate-specific germ cell marker and essential for primordial germ cell survival in orange-spotted grouper [61]. Dmrt1 plays an important part in sex determination and is restricted specifically to spermatogenic cells in orange-spotted grouper [62,63]. Oct4 and Gfra1 critically participate in SSC self-renewal [3,64], and can be detected in medaka SG3 [26,65] and cultured tilapia SSCs [66]. C-kit, a tyrosine kinase receptor, plays an important role in spermatogenesis [67], as well as exists in medaka SG3 [26] and cultured carp SSCs [23]. Under a culture condition being lacking bFGF, LIF, and SCF, GPT cells did not transcribe the marker genes except for *cxcr4b* and *dazl*. SCF addition could induce GPT cells to express several marker genes, *plzf*, *cxcr4b*, *dazl*, *vasa*, *piwi*, and *dnd* at a very low level. GPT cells co-stimulated by LIF and bFGF would obviously increase the mRNA expression of genes, *plzf*, *nanog*, *cxcr4a*, *cxcr4b*, *dmrt1*, *dazl*, and *gfra1*, whereas the expression of *vasa*, *dnd*, *nanos2*, and *oct4* were slightly increased. It must be pointed out that the expression of *piwi* was very faint under any cytokines combinations and even undetectable in some cases. Intriguingly, the expressions of *oct4* and *gfral* were induced in GPT cells mainly by LIF and bFGF. Except for *plzf* and *nanog*, other genes were slightly or absently detected in the rest combinations of cytokines. The differentiated spermatogonia marker gene *c-kit* was not detected in GPT cells under any conditions examined in this study. Apparently, the combination of bFGF and LIF allowed the GPT cells to express more germ cell marker genes at a higher level.

### 3.4. Meiotic Potency of GPT Cells

**Figure 5:**
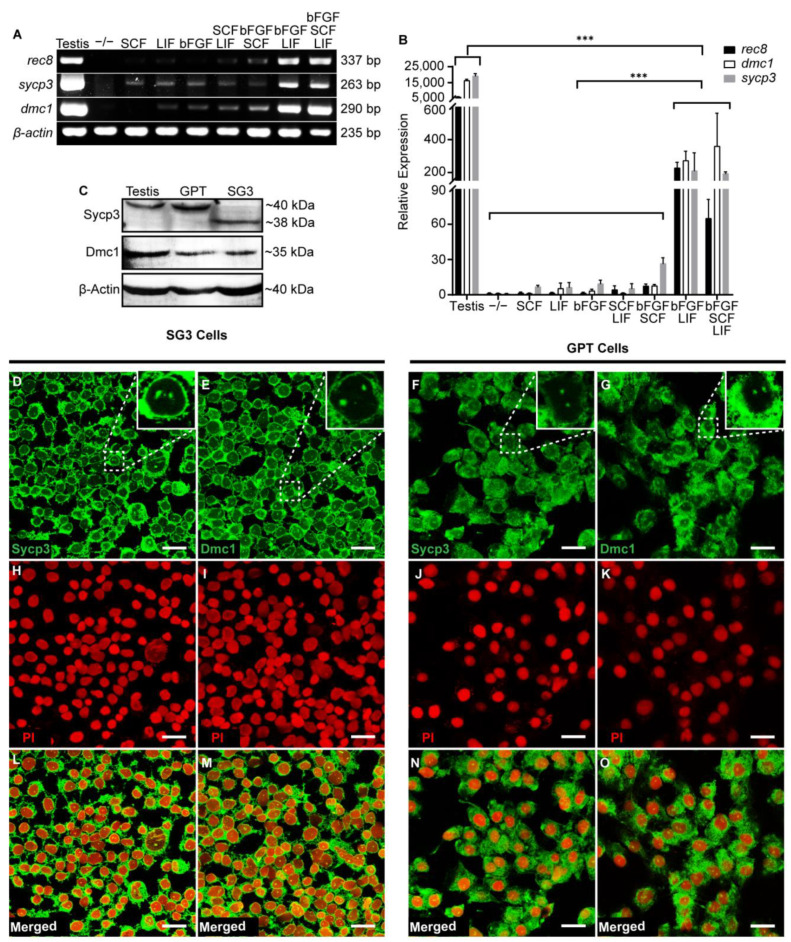
Expression analysis of meiotic markers in GPT cells. (**A**) RT-PCR analysis of the effect of different cytokine combinations on the expression of *rec8*, *sycp3*, and *dmc1* in GPT cells. (**B**) RT-qPCR analysis of the effect of different cytokine combinations on the expression level of *rec8*, *sycp3*, and *dmc1* in GPT cells. The data were shown as the mean ± SEM of three samples and the values with asterisks were significantly different (*** *p* < 0.001). Statistical analysis was performed by one-way ANOVA and Student’s *t*-test. The *β-actin* was used as an internal control. (**C**) Protein expression of Sycp3 and Dmc1 in grouper testis, as well as the GPT cells and SG3 cells treated with bFGF and LIF. The β-Actin was used as an internal control. (**D**–**G**) Fluorescent immunostaining of Sycp3 and Dmc1 in the SG3 cells and GPT cells treated with bFGF and LIF. (**H**–**K**) Nucleus was counterstained with PI. (**L**–**O**) Merge images. Scale Bars: 20 μm.

The meiotic potential of GPT cells was determined through examining the expression and subcellular localization of related meiotic-specific marker genes. The initiation of meiotic onset requires the pre-meiosis marker Rec8, a meiotic recombination protein [68], and the early meiotic marker Sycp3, a component of the synaptonemal complex [69]. Rec8 is restricted to spermatogonia and spermatocytes in the testis of orange-spotted grouper [70]. Dmc1 promotes homologue recombination during early meiotic prophase [71]. Under the co-stimulation of bFGF and LIF, GPT cells were capable of clearly expressing the meiotic-specific marker genes *rec8*, *sycp3*, and *dmc1* (Figure 5A). RT-qPCR analysis indicated that the mRNA expression levels of *rec8*, *sycp3*, and *dmc1* in GPT cells were less than that in testis (Figure 5B). Sycp3 was detected as a band of about 40 kDa in grouper testis and GPT cells, and a band of about 38 kDa in the medaka SG3 cells (Figure 5C). Dmc1 was detected as a distinct band of about 35 kDa in grouper testis, GPT cells, and SG3 cells respectively (Figure 5C). The fluorescent immunostaining analysis in the adult testis of grouper clearly showed that the Sycp3 and Dmc1 antibodies were able to specially mark germ cells out (Figure S2N,O). Additionally, the dot signals of Sycp3 in the nuclei of germ cells in grouper testis are consistent with that in zebrafish [40]. Furthermore, immunocytofluorescence revealed that the Sycp3 and Dmc1 signals were found in the cytoplasm of SG3 cells and GPT cells, and showed multiple bright particles in their nuclei (Figure 5D–G). Nuclei of SG3 cells and GPT cells were counterstained with PI (Figure 5H–K). Merge images were shown in Figure 5L–O. Therefore, the Sycp3 and Dmc1 antibodies could be used for examining the expression of the Sycp3 and Dmc1 proteins in grouper germ cells.

**Figure 6:**
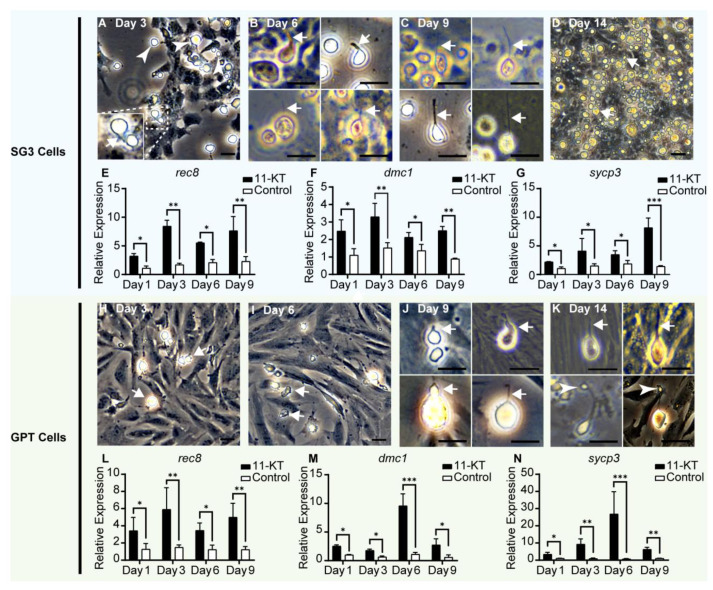
In vitro differentiation of SG3 cells and GPT cells through 11-KT induction. (**A**) SG3 cells produced spherical cells with diverse sizes (Arrowheads) and a few spherical cells with a short protrusion (Arrows) after 3 days of induction. (**B**) A few spherical cells further elongated their tails like the sperm (Arrows) after 6 days of induction. (**C**) About 9 days, a few sperm-like cells had a long tail (Arrows). (**D**) SG3 cells produced a large number of spherical cells and a few sperm-like cells with a tail (Arrows) about 14 days of treatment. (**E**–**G**) The expressions of meiotic marker genes *rec8*, *dmc1*, and *sycp3* in SG3 cells were significantly promoted by 11-KT induction. With the elongation of 11-KT induction time, the expression level of genes, except *sycp3*, did not show an increasing trend. The data were shown as the mean ± SEM of three samples and the values with asterisks were significantly different (* *p* < 0.05, ** *p* < 0.01, and *** *p* < 0.001). Statistical analysis was performed by one-way ANOVA and Student’s *t*-test. (**H**) GPT cells generated a few spherical cells with diverse sizes (Arrows) after about 3 days of induction. (**I**) Some spherical cells gradually died out (Arrows) about 6 days of induction. (**J**) A few spherical cells could grow a short protrusion (Arrows) about 9 days of treatment. (**K**) About 14 days of induction in GPT cells, only a small minority of spherical cells could elongate their protrusions like the sperm-like cells (Arrows). And several sperm-like cells were observed as a sperm head-like ball and a residual body (Arrowheads) connected by a threadlet. (**L**–**N**) The expressions of meiotic marker genes, such as *rec8*, *dmc1*, and *sycp3* in GPT cells were significantly promoted by 11-KT induction. After 6 days of 11-KT induction, the expression level of *dmc1* and *sycp3* reached the highest level, and then became decreased, whereas the *rec8* expression did not show a distinct change in expression level. The data were shown as the mean ± SEM of three samples and the values with asterisks were significantly different (* *p* < 0.05, ** *p* < 0.01, and *** *p* < 0.001). Statistical analysis was performed by one-way ANOVA and Student’s *t*-test. Scale Bars: 20 μm.

Androgen 11-KT was employed to induce the in vitro meiosis of GPT cells and SG3 cells. SG3 cells gradually generated some spherical cells with diverse sizes about 4 to 15 μm in diameter, during 3 days of 11-KT treatment (Figure 6A). Interestingly, we observed a few spherical cells with a short protrusion. About 6 to 9 days of 11-KT treatment on SG3 cells, the cells with a short protrusion would further elongate their protrusions like an elongating spermatid (Figure 6B,C). The sperm-like cells possessed different sizes of sperm head-like balls about 4 to 15 μm in diameter. About 14 days of 11-KT treatment, lots of spherical cells detached from SG3 cells, but only several sperm-like cells with a tail were observed (Figure 6D). During 9 days of 11-KT treatment, SG3 cells significantly expressed *rec8*, *sycp3*, and *dmc1* (Figure 6E–G). During 3 to 6 days of 11-KT treatment, GPT cells produced only a few spherical cells with diverse sizes about 5 to 18 μm in diameter (Figure 6H), and some of them gradually disappeared (Figure 6I). After 9 days of induction, a few spherical cells with a short protrusion were observed (Figure 6J). Similar to SG3 cells, GPT cells could also generate a few sperm-like cells with a sperm head-like ball and a tail after about 14 days of 11-KT induction (Figure 6K). Occasionally, several sperm-like cells with a putative residual body were observed (Figure 6K). Likewise, 11-KT treatment could induce GPT cells to express *rec8*, *sycp3*, and *dmc1* at a higher level (Figure 6L–N). In a word, the GPT cell line is capable of responding to the 11-KT stimulation and in vitro differentiating into sperm-like cells.

## 4. Discussion

The SSC line, a valuable stem cell system to investigate germ cell biology and spermatogenesis, can be used for genetic manipulation and germ cell transplantation. However, the fish SSC line is hard to be established and reported limitedly. Especially, the SSC line is not yet obtained and documented in hermaphroditic vertebrates and marine fish. Here, we successfully established an SSC line being in vitro propagated for more than 20 months and 120 passages, designed as GPT, from the adult testis tissue of a protogynous hermaphroditic fish, orange-spotted grouper.

As previous studies reported [14,26], cultured SSCs have a round or polygonal phenotype, a large nucleus, alkaline phosphatase activity, and the gene expression pattern of germ cells. Similarly, GPT cells had a polygonal cell shape, a large nucleus with obvious nucleoli, and high alkaline phosphatase activity. A frequently adopted criterion to identify the putative germ stem cells is to examine whether they are capable of expressing the germ cell marker and the pluripotency marker [72]. Primarily, GPT cells expressed the well-known germline markers, *vasa*, Dazl, and Piwi [48]. Other germ cell marker genes also were detected in GPT cells, such as *ly75*, *dnd*, *oct4*, and *nanog* [61,73]. Additionally, the expression of pluripotency markers Ssea1, Nanog, *gfra1*, and *oct4* further indicates that GPT cells possess stem cell pluripotency. Because *plzf*, *zbtb40*, and *dmrt1* are male germ cell-specific genes [49,62,63], GPT cells are genetically masculinized and do not originate from the latent female germ cells of testis. In vitro culture leads to a significant change in gene expression pattern of SSCs. GPT cells after long-term in vitro culture, exhibit some deficiencies, such as the weaker expression of *piwi* and *oct4*, and the absence of *c-kit* transcript, compared with those in the in vivo gonadal cells. The similar situations could be found in the SSCs culture system of other animals. The SSC line of hook snout carp expresses *dmrt1* at an almost undetectable expression level [27]. Porcine (*Sus scrofa*) SSCs under culture conditions could only weakly express *plzf*, *c-kit*, and *nanog* [52]. Therefore, the absence or weaker expression of a few marker genes does not negate the identification of SSCs. The putative SSCs should be identified from multiple perspectives, including, but not limited to, stem cell pluripotency, meiotic ability, and germ cell markers. Under the normal culture condition, mouse SSC lines can both maintain self-renewal, and have the potential to spontaneously produce the differentiated germ cells with the expression of meiotic marker genes, such as *stra8* and *sycp1* [10,15]. Similarly, *dmc1* or *sycp3* could be detected in the SSCs culture systems of medaka and zebrafish [26,40]. Thus, the expression of genes related to spermatogenesis in SSC lines is indicative of meiotic potential of cells. In orange-spotted grouper, expression levels of *sycp3* and *rec8* are upregulated markedly when germ cells start meiotic onset [74]. The expression level of *dmc1* is also enhanced when SG3 cells begin spermatogenesis [26]. When incubated with bFGF and LIF, or 11-KT, GPT cells were capable of significantly transcribing *rec8*, *sycp3*, and *dmc1*. Moreover, localization patterns of Sycp3 and Dmc1 in GPT cells were consistent with SG3 cells and the male germ cells of grouper testis. Therefore, it is deduced that GPT cells possess the potential to start meiotic onset in vitro. In a word, GPT cells exhibit germline characteristics, including SSC phenotypic traits, the expression of germ cell markers, as well as the ability to begin meiotic onset.

The cytokine bFGF was indispensable to the long-period cultivation of GPT cells. Nevertheless, its shortcomings in spermatogonial properties could not be ignored, including over-low expressions of some germ cell marker genes, such as *vasa* and *piwi*, as well as differentiation into many epithelial-like cells. Therefore, bFGF is necessary but not enough to sustain the self-renewal of GPT cells. SCF neither reduced the potency of cell differentiation nor significantly promoted the expression of germ cell markers in GPT cells. Likewise, we discovered that GDNF addition could not affect GPT cells in cell morphology and gene expression (not shown data). In the SSCs culture systems of medaka, mouse, and sturgeon (*Acipenser dabryanus*), LIF can promote cell proliferation and self-renewal [16,75,76]. Unexpectedly, after the bFGF and LIF treatment, GPT cells can recover spermatogonia traits and pluripotency just to some extent. These rehabilitative changes include a cytomorphology looking more like cultured SSCs, a higher expression level of the germ cell and meiosis markers, reappearances of the lost germ cell marker genes *oct4* and *gfra1*, and the potential for generating diverse phenotypic cells. Gfra1 is both a receptor of GDNF and a surface marker of SSCs in mammals and fish [6,13,72]. Interestingly, LIF was able to stimulate GPT cells to express *gfra1* in the absence of GDNF. Confusedly, under the co-stimulation of bFGF and LIF, GPT cells were capable of highly transcribing the early meiotic genes *rec8*, *sycp3*, and *dmc1*. According to that, under a culture condition, SSCs of some fish species would spontaneously start spermatogenesis and generate sperm in vitro [23,39,40], we conjecture that GPT cells after recovering some spermatogonia traits might have a stronger intrinsic ability to initiate meiosis autonomously. In brief, the GPT cells with the induction of bFGF and LIF would regain some spermatogonial properties, maintain self-renewal, and possess the ability of meiosis initiation in vitro.

In fish, 11-KT is important for spermatogenesis and can be employed to induce in vitro spermatogenesis [36,37,77]. SG3 cells are capable of generating motile sperm by high cell confluence without subculture [26]. However, via this method, GPT cells only produced a few spherical cells (Figure S5). When incubated with bFGF and LIF, GPT cells occasionally produced a few spherical cells of different sizes. Whereas, 11-KT improved the expression levels of *rec8*, *sycp3*, and *dmc1* in GPT cells and prompt the cells to stably produce a few spherical cells and sperm-like cells. Similar results were also obtained in the SG3 cells treated with 11-KT. The spherical cells from SG3 cells and GPT cells were in different sizes, implying that the small spherical cells might be divided from the large spherical cells. In induced differentiation of GPT cells, we observed several sperm-like cells possessing a putative residual body, which could also be produced by SG3 cells [26]. In addition, we found that another androgen, 17 alpha methyl testosterone, could also promote the differentiation of sperm-like cells from SG3 cells and GPT cells (not shown data). In zebrafish testis, androgen receptor is restricted to the Sertoli cells surrounding SSCs [78]. Nonetheless, the zebrafish lacking androgen receptor could produce fertile sperm [79]. In eels, androgen receptor is expressed in both Sertoli and male germ cells [80]. Similarly, androgen receptor is also expressed in Sertoli cells and male germ cells in orange-spotted grouper (data not shown). In this study, with the absence of Sertoli cells, both SG3 cells and GPT cells were able to produce sperm-like cells after the 11-KT treatment, suggesting that 11-KT probably acts directly on SSCs to promote spermatogenesis without Sertoli cell mediation in fish. Nonetheless, we did not observe the increment of sperm-like cells and the appearance of fully developed and motile sperm-like cells, even prolonging the induction and increasing the dosage of 11-KT (data not shown). We speculate that 11-KT might initiate, but not be insufficient for completing the process of spermatogenesis of GPT cells in vitro.

When the SSCs with the fluorescent labeling are transplanted into an embryo, larva, and mature gonads, they can be in vivo traced for exploring the differentiation of SSCs [66,81,82]. Due to the lack of the spermatocyte and spermatid markers in grouper, and the scarcity and incomplete development of sperm-like cells, it is difficult to further examine the in vitro spermatogenesis of GPT cells. To verify the differentiation potency of GPT cells by cell transplantation technique in the future, we established a GPT cell line stably expressing green fluorescence protein using electrotransfection with a pEGFP-N3 plasmid (Figure S6). It is particularly interesting to investigate whether GPT cells can be used for generating genetic modified offspring. The behaviors of GPT cells in recipient gonads might offer new insights into the understanding of gametogenesis and sex reversal in hermaphroditic vertebrates.

## 5. Conclusions

In the present study, we successfully established an SSC line from adult testis of a protogynous hermaphroditic fish, orange-spotted grouper. The grouper SSC line expressed a series of germ cell markers, retained in vitro proliferation over 20 months in the effect of bFGF and LIF, and produced sperm-like cells under the 11-KT treatment. To our knowledge, it is the first report about the establishment of an SSC line in hermaphroditic vertebrates and marine fish. The grouper SSC line, as a unique stem cell system, can be applied in studying reproductive physiology in hermaphroditic vertebrates. Since grouper is a large and farmed marine fish, its SSC line should have great value and potential applications in developing related techniques of fish breeding.

## Figures and Tables

**Table 1:** Primers for RT-PCR and in situ hybridization (ISH).

Gene	Primer (5′ to 3′)	Product Size
*vasa*	F: GCTGATTTCATCGCCACTTAT R: CGTAGAAAGACACCGCCCTC	277 bp
*piwi*	F: GTGAAGAAGGTGGGTCCTGTTG R: CATTGGCATTACGATGGGTGT	424 bp
*dazl*	F: GCAACAGATCCGATTTAAGGG R: GGTTCATTGGCATAGGTGGG	254 bp
*nanos2*	F: ACTACCCTCTCCGGGACTATGT R: GCGTCATCAGTCATTTCACTTTCCC	348 bp
*dnd*	F: GGCCCGCTGTGAAGTCTTCATCAG R: TTGCCATTGAAGCAGCATAGTGGG	419 bp
*plzf*	F: CAGCCCTGGCAATGTCTATG R: TCTGGCGGGATGTCTTCG	904 bp
*nanog*	F: GGCAACAACAAAGGAAGCCCAATT R: TAGCCAGCAGGTCCACCAGCAGAG	197 bp
*oct4*	F: TCTACAACAAACCCGCTTACAGT R: GCAGAACCAAACACGAACGAC	1037 bp
*gfra1*	F: ACGCCGACGACAAACTAT R: CCACGGTGACAGGCTAAT	268 bp
*zbtb40*	F: AGAAACCGTTTGCTTGCG R: TCCACCTGACAGAGCCACA	1055 bp
*ly75*	F: AGACATCCTAACCATCCGAAAT R: AGTAAGCCAGAGCCGAGCC	1052 bp
*thy1*	F: AAGCCCAAAGCCAACAAG R: CAAACGATCCAGGAGCAG	311 bp
*dmrt1*	F: CCCGCTGTAGAAACCACGGCTAT R: GGTCCGACTGTGCGTCAGTATGAG	369 bp
*c-kit*	F: CACCAAGCCCACCATTACC R: TGAAGCCTTTCTCATAAACATCG	864 bp
*cxcr4a*	F: CTGGTCCGAGCAGTTAGAG R: TCAAGTTCACAAGAGGGAGA	943 bp
*cxcr4b*	F: CTGGGCATCACTGGAAACG R: GGCTCTGCGTGCTGAACTCTT	831 bp
*rec8*	F: ACCGCAACCCAACATACCGA R: GGAGTGCTGTGAACCTGCCTCT	337 bp
*sycp3*	F: CAGCATTGGGAGACTGAAGC R: TGTTGCGTGTCCATGAGGAT	263 bp
*dmc1*	F: TCCAAGACATTGACCTCCTA R: CTCTATACCGCCACCTAAAA	290 bp
*amh*	F: TTGGCGTTTGAAAGTCCG R: GATGTTGGCAGTGTTTGGTC	894 bp
*sdf1*	F: ACAAGCAAAGCCCATCAGTC R: TGTTAATGGCGTTCTTCAGGT	206 bp
*cyp11b2*	F: GAGCGGCTGGGTCAACTT R: GCCACTCCTCACCGTTCTTG	275 bp
*β-actin*	F: TTCACCACCACAGCCGAGA R: TGGTCTCGTGGATTCCGCAG	235 bp

**Table 2:** RT-qPCR primers for examining meiotic marker genes’ expression in the testis cells of orange-spotted grouper.

Gene	Primer (5′ to 3′)	Product Size
*rec8*	F: CACTCCTGCCAGCAGATGGTC R: GACCTCTCCAAACCTCTGCA	157 bp
*sycp3*	F: AGTGGTGCAGAACCAGAAACTG R: TGTTGCGTGTCCATGAGGAT	168 bp
*dmc1*	F: TGCTGGACAACGTGCTTTAC R: AGTTTCTGCTGCCGCTCA	187 bp
*β-actin*	F: AAATCGCCGCACTGGTTGTT R: CCCTCTTGCTCTGGGCTTCAT	177 bp

**Table 3:** RT-qPCR primers for examining meiotic marker genes’ expression in SG3 cells.

Gene	Primer (5′ to 3′)	Product Size
*rec8*	F: CCCTGTTCCCTCCGATAAAGA R: TCCTGCGGTCCACATTCG	179 bp
*sycp3*	F: TTTAGTGGCGGGAAGACG R: GCACATTCATCCGCTCCTT	148 bp
*dmc1*	F: TGGCGCTGTTCAGAGTGG R: CGATGGGCTTCTTGGGAT	189 bp
*β-actin*	F: GCTGGATTCGCTGGAGACG R: CAATGGGATACTTCAGGGTCAG	160 bp

## Data Availability

The study did not report any other data.

## Supplementary Materials

The following supporting information can be downloaded at: https://www.mdpi.com/article/10.3390/cells11182868/s1, Figure S1: Cell localization of *ly75*, *thy1*, and *dmc1* in adult testis of orange-spotted grouper; Figure S2: Fluorescent immunostaining of antibodies in adult testis of orange-spotted grouper; Figure S3: Derivation of a single colony from a single cell in GPT line; Figure S4: Prolonged cultivation of GPT cells under the lack of bFGF; Figure S5: Morphology of GPT cells under a condition of high cell confluence; Figure S6: Establishment of a GPT cell line stably expressing green fluorescence protein.
