# Supplementary material for: Establishment of a Spermatogonial Stem Cell Line with Potential of Meiosis in a Hermaphroditic Fish, Epinephelus coioides
Source: Cells. 2022 Sep 14;11(18):2868. doi: 10.3390/cells11182868 (PMC9496998; doi:10.3390/cells11182868)
Supplement: Supplementary file 1 [file cells-11-02868-s001.zip › cells-1858742-supplementary.pdf]

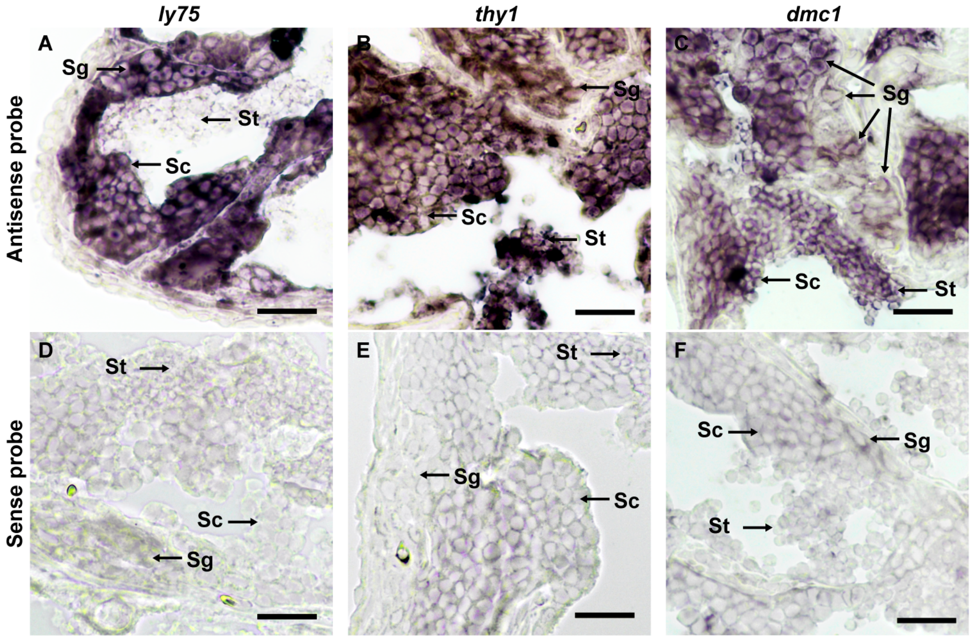

Figure S1: Cell localization of *ly75*, *thy1*, and *dmc1* in adult testis of orange-spotted grouper. (**A**–**C**) Antisense probe signals of *ly75*, *thy1*, and *dmc1*. (**D**–**F)** Sense probe signals of *ly75*, *thy1*, and *dmc1*. Sg, Spermatogonium; Sc, Spermatocyte; St, spermatid. Scale Bars: 20 μm.

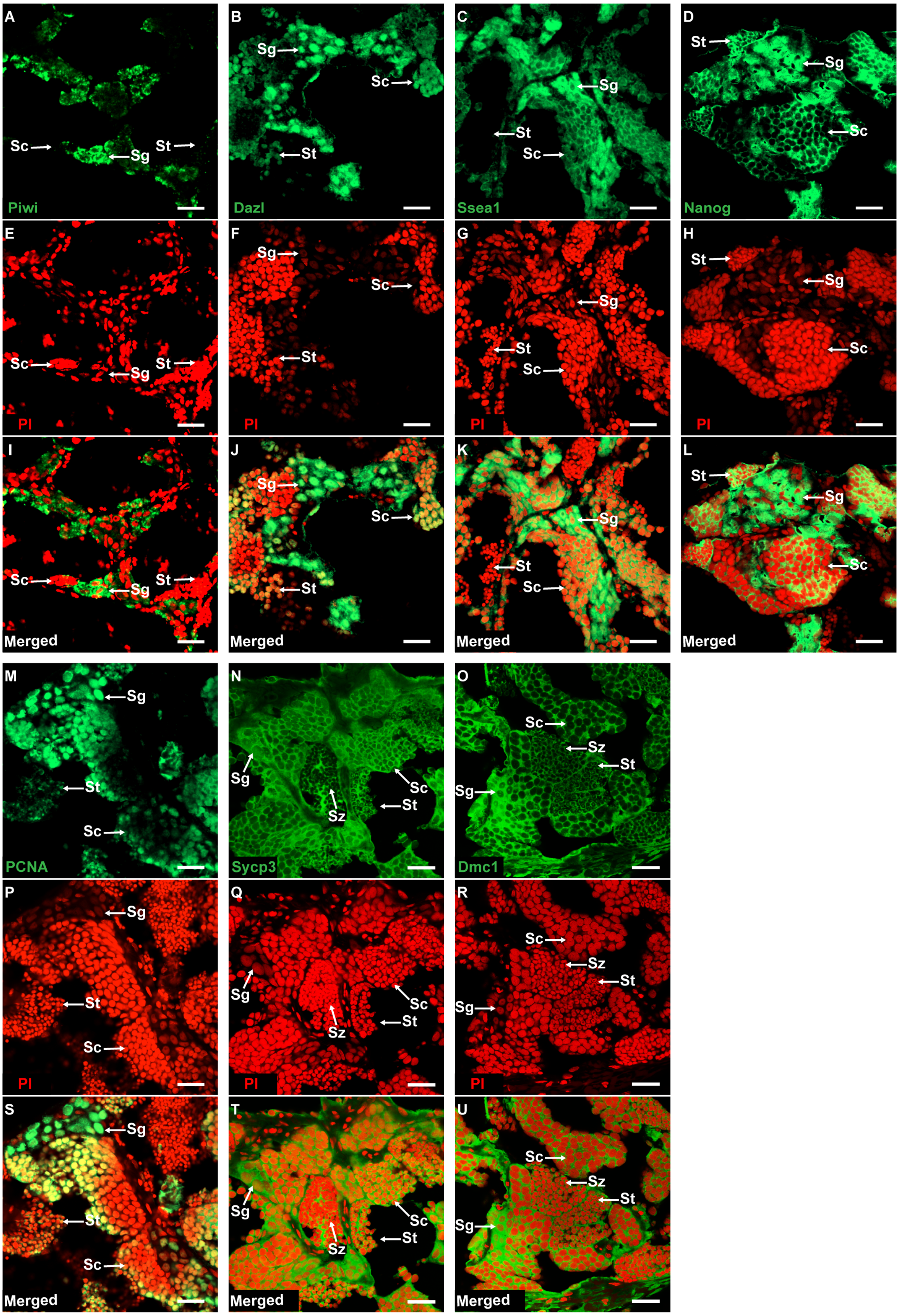

Figure S2: Fluorescent immunostaining of antibodies in adult testis of orange-spotted grouper. (**A–D** and **M–O**) Fluorescence signals of Piwi, Dazl, Ssea1, Nanog, PCNA, Sycp3, and Dmc1. (**E–H** and **P–R**) Nuclei are counterstained with PI. (**I–L** and **S–U**) Merge images. Sg, Spermatogonium; Sc,

Spermatocyte; St, spermatid; Sz, spermatozoa. Scale Bars: 20 μm.

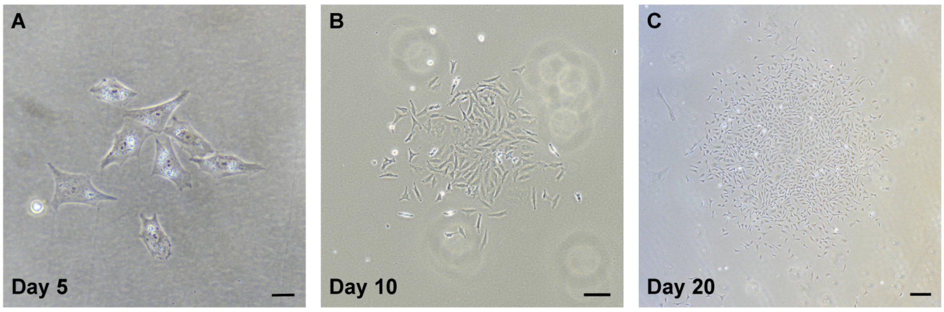

Figure S3: Derivation of a single colony from a single cell in GPT line. (**A**) A small colony after five days of culture. (**B**) A distinct colony after 10 days of culture. (**C**) A large colony containing hundreds of cells after 20 days of culture. Scale Bars: 20 μm in A; 50 μm in B; 200 μm in C.

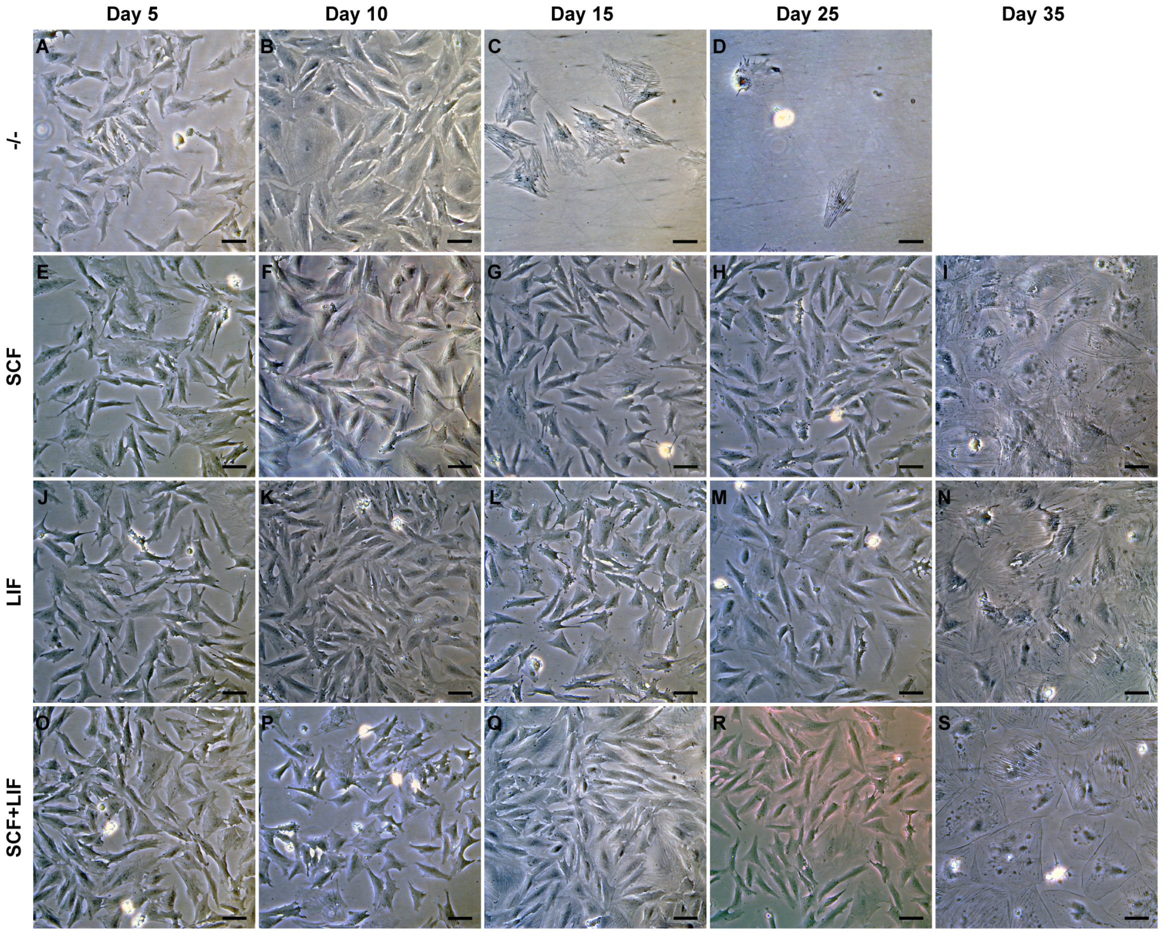

Figure S4: Prolonged cultivation of GPT cells under the lack of bFGF. (**A–D**) All GPT cells would differentiate into large epithelial-like cells and gradually die out during 25 days of culture under the lack of bFGF, LIF, and SCF (−/−). (**E–H**, **J–M**, and **O–R**) GPT cells consisted of some polygonal-like cells and many epithelial-like cells during 25 days of culture in the ESM media containing SCF and/or LIF. (**I**, **N** and **S**) All GPT cells transformed into very large epithelial-like cells and gradually died after 35 days of culture in the ESM media containing SCF and/or LIF. Scale Bars: 50 μm.

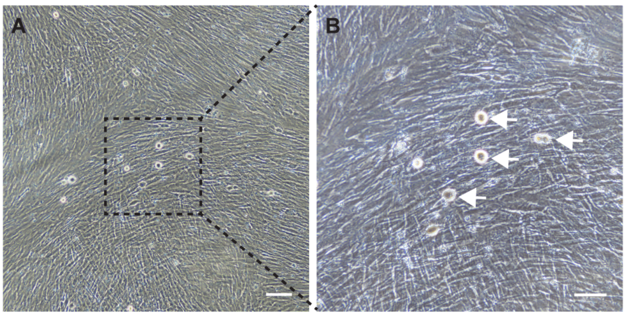

Figure S5: Morphology of GPT cells under a condition of high cell confluence. (**A**) GPT cells were cultured for 14 days without subculture and (**B**) generated a few spherical cells (Arrows). Scale Bars: 50 μm in A; 20 μm in B.

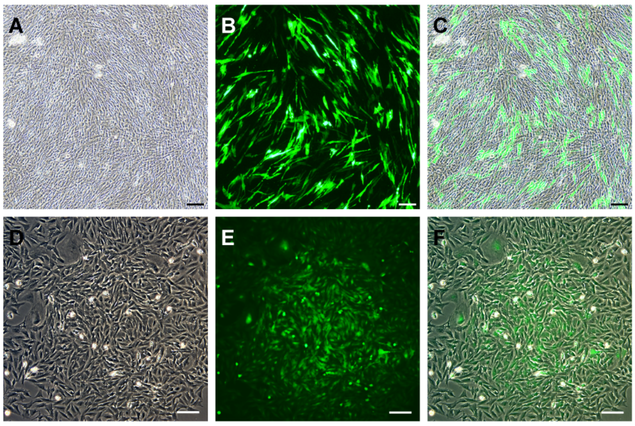

Figure S6: Establishment of a GPT cell line stably expressing green fluorescence protein. (**A**–**C**) Bright-field image, fluorescent image, and merged image of GPT cells after 48 hours of culture following electrotransfection with pEGFP-N3 plasmid. (**D**–**F**) Bright-field image, fluorescent image, and merged image of GPT cells after G418 resistance screening. Scale Bars: 200 μm in A–C; 100 μm in D-F.
